# The Prevalence and Factors Associated With Anxiety-Like and Depression-Like Behaviors in Women With Polycystic Ovary Syndrome

**DOI:** 10.3389/fpsyt.2021.709674

**Published:** 2021-10-20

**Authors:** Han Lin, Mingxing Liu, Dongmei Zhong, Ernest Hung Yu Ng, Jianping Liu, Juan Li, Yu Shi, Chunren Zhang, Xiaohui Wen, Zhefen Mai, Miaoxian Ou, Hongxia Ma

**Affiliations:** ^1^Department of Gynecology of Traditional Chinese Medicine, The First Affiliated Hospital of Guangzhou Medical University, Guangzhou, China; ^2^Department of Obstetrics and Gynecology, The Third Affiliated Hospital of Guangzhou Medical University, Guangzhou, China; ^3^Department of Gynecology of Traditional Chinese Medicine, the Third Affiliated Hospital of Guangzhou Medical University, Guangzhou, China; ^4^Department of Obstetrics and Gynecology, the University of Hong Kong, Hong Kong Special Administrative Region, Hong Kong, Hong Kong, SAR China; ^5^Centre for Evidence-Based Chinese Medicine, Beijing University of Chinese Medicine, Beijing, China; ^6^Guangzhou University of Chinese Medicine, Guangzhou, China

**Keywords:** PCOS, anxiety, depression, hyperandrogenism, metabolic syndrome

## Abstract

Increasing evidence shows that polycystic ovary syndrome (PCOS) patients are particularly vulnerable to anxiety/depression-like behaviors. This study sought to determine the prevalence of anxiety/depression-like behaviors among women with PCOS and to identify factors associated with these behaviors. This study was a secondary analysis of three studies performed on Chinese women who were aged 18 to 40 and diagnosed with PCOS according to the modified Rotterdam criteria. We obtained 802 useable responses for the self-rating anxiety scale and 798 responses for the self-rating depression scale. The prevalence of anxiety-like and depression-like behaviors among women with PCOS was 26.1% (209/802) and 52.0% (415/798), respectively. Anxiety-like behaviors were associated with age, body image-related factors (including body mass index and waist-to-hip ratio), and hyperandrogenism-related factors (including free androgen index and hirsutism). Depression-like behaviors were associated with age, body image-related factors, hyperandrogenism-related factors, and metabolic factors (including fasting insulin, fasting plasma glucose, and homeostatic model assessment of insulin resistance). Body image-related factors and hyperandrogenism-related factors were related to both anxiety-like behaviors and depression-like behaviors in both infertile and fertile PCOS patients.

## Introduction

Polycystic ovary syndrome (PCOS) is a chronic gynecological endocrine and metabolic disease affecting about 6–10% of women of childbearing age worldwide (1). The etiology of PCOS mainly involves hormone imbalances that lead to irregular menstruation and to ovulation dysfunction (1). In addition to these symptoms, classic clinical symptoms of PCOS also include clinical or laboratory evidence of elevated androgens and polycystic ovarian morphology as shown by pelvic ultrasound (2). Compared to healthy women, women with PCOS are at greater risk for morbidities and complications in other systems, including diabetes, dyslipidemia, hypertension, obesity, metabolic syndrome, obstructive sleep apnea, pregnancy complications, endometrial cancer, and cardiovascular disease (3–8).

In addition to all of the long-term metabolic and endocrine outcomes, an increased prevalence of psychological disorders among women with PCOS has also been reported in some studies, including depression and anxiety (9–12). It has been reported in some studies that women with PCOS suffer from more mental stress than healthy women, with many of them reporting dissatisfaction with their body image or acne on their face or worry about their infertility (13, 14). These factors associated with PCOS make these women more vulnerable to feeling anxious and upset, but the specific causative factors, including sociological factors and molecular mechanisms, for these anxiety/depression-like behaviors in women with PCOS have not been fully elucidated.

The most common mental disorders studied in women with PCOS are anxiety and depression (15), and these usually result from changes in their body image as a result of their disease (including hirsutism, irregular menses, obesity, acne, and hair thinning) (16). The self-rating anxiety scale (SAS) and the self-rating depression scale (SDS) are commonly used for self-evaluation of the presence and severity of anxiety-like and depression-like behaviors (17–19).

In this study, we obtained a total of 802 SAS scores and 798 SDS scores after excluding those with a completion rate below 50% from the 828 women with PCOS in our three studies. By analyzing the relationship between SAS/SDS score and other baseline data, we have identified the most likely factors associated with anxiety-like and depression-like behaviors in these women.

## Materials and Methods

### Overview and Participants

This study was a retrospective secondary analysis of women with PCOS from three trials, including one prospective pilot trial and two randomized controlled trials. The study protocol has been described in *The Effect of Acupuncture on Insulin Sensitivity Polycystic Ovary Syndrome* (ClinicalTrials.gov NCT 02026323) (20), *The Effect of Acupuncture on Insulin Sensitivity of Women with Polycystic Ovary Syndrome and Insulin Resistance: a Randomized Controlled Trial* (ClinicalTrials.gov, NCT 02491333) (21), and *The Effect of Acupuncture Pre-treatment Combined with Letrozole on Live Birth in Infertile Women with Polycystic Ovary Syndrome: a Randomized Controlled Trial* (ClinicalTrials.gov NCT 02491320) (22). The ethics approvals were granted from the ethics committee of the First Affiliated Hospital of Guangzhou Medical University (ref. 2013039, ref. 2015010, ref. 2014018, respectively), as is shown in detail in Supplementary 1. These three trials were conducted in the Department of Traditional Chinese Medicine of the First Affiliated Hospital of Guangzhou Medical University, the Department of Gynecology of Xuzhou Maternity & Child Health Hospital, the Department of Traditional Chinese Medicine of Dalian Municipal Women and Children's Medical Center, the Department of Gynecology of Guangdong Women and Children's Hospital, and the Department of Traditional Chinese Medicine of Hexian Memorial Affiliated Hospital of Southern Medical University. The three studies involved a total of 828 Chinese women aged 18–40 years who met the PCOS diagnosis criteria listed, and in this study the included participants had also completed both the SDS and SAS.

### Selection Criteria

Inclusion criteria:

1. Women aged between 18 and 40 years.
2. Body mass index (BMI) ≥ 18.5 kg/m^2^.
3. Diagnosis of PCOS as defined by the Rotterdam criteria (Supplementary 2).
4. Willingness to sign the consent form.
5. A completion rate above 50% for the SAS and SDS.

Exclusion criteria:

1. A completion rate below 50% for the SAS and SDS.
2. Other endocrine disorders, as shown in Supplementary 2.
3. Use of hormones or other medications, including Chinese herbal prescriptions, that might affect the outcome in the past 2 months.
4. Receiving acupuncture in the past 2 months.
5. Pregnancy within the past 6 weeks.
6. Abortion or having given birth within the past 6 weeks.
7. Breast feeding within the past 6 weeks.
8. Not willing to give written consent to participate.
9. Having a bariatric surgery procedure within the past 12 months or being in a period of acute weight loss.
10. Additional exclusion criteria as shown in Supplementary 2.

### SAS/SDS Standard Score Calculation and Grading

Both the SAS and SDS have 20 items, each of which is graded on a scale of 1–4. Adding all the scores of each question in the SAS or SDS gives a raw score with a minimum of 20 points and a maximum of 80 points, and this is then converted into a standard score. For the SAS, the standard score is equal to the raw score × 1.25 (rounded to the nearest whole number). In the general Chinese population an SAS standard score (SAS-SS) ≥50 is considered to be an indication of anxiety, with scores of 50–59 classified as mild, 60–69 classified as moderate, and ≥70 classified as severe (19). The standard score for the SDS (SDS-SS) is calculated the same as for the SAS-SS. For the general Chinese population, an SDS-SS ≥53 is regarded as a depression-like state, with scores of 53–62 classified as mild, 63–72 classified as moderate, and ≥73 classified as severe (19).

### Analysis of Factors Associated With Anxiety/Depression-Like Behaviors in Women With PCOS

The clinical data included BMI, waist circumference (WC), waist-to-hip Ratio (WHR), acne, testosterone (T), hirsutism (Ferriman–Gallwey Score), sex hormone-binding globulin (SHBG), free androgen index [FAI, calculated as T (nmol/L)^*^100/SHBG (nmol/L) (23)], the ratio of luteinizing hormone to follicle-stimulating hormone (LH/FSH), fasting insulin, fasting plasma glucose (FPG), homeostatic model assessment of insulin resistance [HOMA-IR, calculated as FPG (mmol/L) ^*^ Fasting Insulin(mIU/L)/22.5 (24, 25)], number of deliveries, and age. We analyzed the correlation between SAS-SS/SDS-SS and these 14 clinical factors by Pearson and Spearman correlation analysis and linear regression analysis in order to determine which factors were independent risk factors for anxiety-like and depression-like behaviors in women with PCOS.

### Subgroup Analysis Between Infertile and Fertile PCOS

Among the 802 participants, there were 369 infertile PCOS patients from *The Effect of Acupuncture Pre-treatment Combined with Letrozole on Live Birth in Infertile Women with Polycystic Ovary Syndrome: a Randomized Controlled Trial* and 433 fertile PCOS patients from the other two RCTs. We performed baseline data analysis, correlation analysis, and linear regression analyses on the two groups of patients separately.

### Statistical Analysis

Descriptive statistics of the data at baseline were analyzed using the chi-square test for categorical variables and using means and standard deviations with Student's *t*-test for continuous variables. The Pearson and Spearman correlation coefficients assessed the association between SAS and SDS parameters and the 14 clinical characteristics. Linear regression analysis assessed the strength of the relationship between SAS-SS or SDS-SS and the 14 baseline characteristics. All statistical analyses were carried out in SPSS 26.0, and *P* < 0.05 was considered statistically significant.

## Results

### Prevalence of Anxiety/Depression-Like Behaviors in Women With PCOS

A total of 802 women with PCOS completed the SAS, and the SAS-SS ranged from 26 to 85. Of these, 26.1% (209/802) had scores ≥50 indicating anxiety-like behaviors, with 21.8% (175/802), 3.9% (31/802), and 0.4% (3/802) showing mild, moderate, and severe anxiety, respectively.

A total of 798 women with PCOS completed the SDS, and the SDS-SS ranged from 25 to 91. Of these, 52.0% (415/798) had scores ≥53 indicating depression-like behaviors, with 37.3% (298/798), 13.4% (107/798), and 1.3% (10/798) showing mild, moderate, and severe depression, respectively.

### Clinical Characteristics

As shown as Table 1, age, BMI, WC, WHR, T, SHBG, and FAI were significantly different (*P* < 0.05) when comparing participants with anxiety (SAS-SS ≥ 50) to those without (SAS-SS < 50). As shown in Table 2, age, BMI, WC, acne, T, FAI, fasting insulin, FPG, and HOMA-IR were significantly different (*P* < 0.05) when comparing participants with depression (SDS-SS ≥ 53) to those without (SDS-SS < 53).

**Table 1:** Baseline characteristics of women with PCOS with anxiety (SAS-SS ≥ 50) and without anxiety (SAS-SS < 50).

**Index**	SAS-SS^*^ ≥ 50 (*N* = 209)	SAS-SS^*^ < 50 (*N* = 593)	***P***
Age	26.46 ± 4.18	27.22 ± 3.84	0.022^*^
BMI	25.18 ± 4.61	24.25 ± 4.44	0.015^*^
WC	85.36 ± 11.34	82.61 ± 11.32	0.004^*^
WHR	0.87 ± 0.066	0.86 ± 0.069	0.039^*^
Acne	48.22% (95/197)	40.46% (229/566)	0.058
T	1.19 ± 0.93	1.70 ± 2.26	0.003^*^
SHBG	38.48 ± 24.08	43.14 ± 29.85	0.046^*^
FAI	3.83 ± 3.43	4.90 ± 6.83	0.034^*^
LH/FSH	1.85 ± 1.10	1.81 ± 1.21	0.697
Number of deliveries			
0	74.62% (147/197)	75.80% (429/566)	0.282
1	21.82% (43/197)	22.61% (128/566)	
2	3.56% (7/197)	1.41% (8/566)	
3	0.00% (0/197)	0.18% (1/566)	
Hirsutism	3.93 ± 3.93	3.43 ± 3.63	0.107
Fasting insulin	17.12 ± 10.29	15.17 ± 12.50	0.055
FPG	5.17 ± 0.47	5.16 ± 0.54	0.880
HOMA-IR	3.98 ± 2.51	3.53 ± 3.04	0.073

*Categorical variables were shown as n% and continuous variables were shown as Mean ± SD*.

*SAS-SS, self-rating anxiety scale standard score; BMI, body mass index; WC, waist circumference; WHR, waist-to-hip Ratio; T, testosterone; SHBG, sex hormone-binding globulin; FAI, free androgen index; LH/FSH, the ratio of luteinizing hormone to follicle-stimulating hormone; FPG, fasting plasma glucose; HOMA-IR, homeostatic model assessment of insulin resistance*.

* *P < 0.05*.

**Table 2:** Baseline characteristics of women with PCOS with depression (SDS-SS ≥ 53) and without depression (SDS-SS < 53).

**Index**	SDS-SS^*^ ≥ 53 (*N* = 415)	SDS-SS^*^ < 53 (*N* = 383)	***P***
Age	27.36 ± 3.76	26.66 ± 4.10	0.017^*^
BMI	24.04 ± 4.37	24.97 ± 4.59	0.005^*^
WC	82.26 ± 10.99	84.43 ± 11.69	0.010^*^
WHR	0.86 ± 0.069	0.87 ± 0.068	0.397
Acne	38.66% (138/357)	46.77% (174/372)	0.027^*^
T	1.85 ± 1.37	1.26 ± 2.48	0.000^*^
SHBG	43.90 ± 28.55	39.91 ± 28.43	0.050
FAI	5.28 ± 4.82	3.95 ± 7.25	0.003^*^
LH/FSH	1.78 ± 1.18	1.87 ± 1.18	0.311
Number of deliveries			
0	77.3% (276/357)	73.4% (273/372)	0.238
1	19.6% (70/357)	24.7% (92/372)	
2	2.8% (10/357)	1.9% (7/372)	
3	0.3% (1/357)		
Hirsutism	3.66 ± 3.86	3.45 ± 3.56	0.463
Fasting Insulin	14.82 ± 9.27	16.57 ± 14.25	0.048^*^
FPG	5.12 ± 0.430	5.22 ± 0.597	0.010^*^
HOMA-IR	3.43 ± 2.28	3.88 ± 3.45	0.037^*^

*Categorical variables were shown as n% and continuous variables were shown as Mean ± SD*.

*SDS-SS, self-rating depression scale standard score; BMI, body mass index; W, waist circumference; WHR, waist-to-hip Ratio; T, testosterone; SHBG, sex hormone-binding globulin; FAI, free androgen index; LH/FSH, the ratio of luteinizing hormone to follicle-stimulating hormone; FPG, fasting plasma glucose; HOMA-IR, homeostatic model assessment of insulin resistance*.

* *P < 0.05*.

### Correlation Analyses Between Anxiety/Depression and Clinical Characteristics

**Figure 1:**
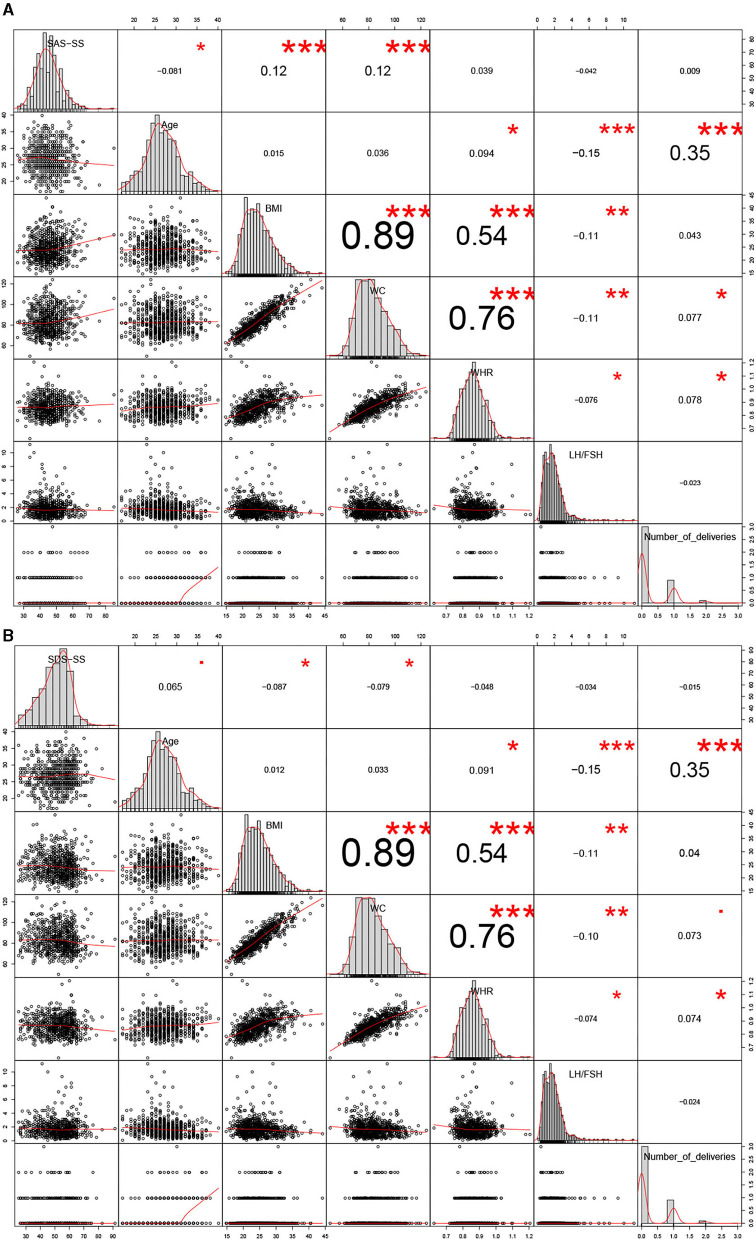
Correlation analyses between anxiety/depression-like behaviors and the 14 clinical parameters. *P < 0.05, **P < 0.01, ***P < 0.005.

We next analyzed the correlation between the severity of anxiety/depression-like behaviors and the baseline clinical characteristics. As shown in Figure 1A, the correlation between age, BMI, and WC and the severity of anxiety was statistically significant and their correlation coefficients were −0.081, 0.12, and 0.12, respectively (*P* < 0.05). BMI and WC were positively correlated with the severity of anxiety, while age was negatively correlated. A shown in Figure 1B, the correlation between age, BMI, W, and the severity of depression was statistically significant, and their correlation coefficients were 0.065, −0.087, and −0.079, respectively (*P* < 0.05). Age showed a positive association with the degree of depression, while BMI and WC showed a negative association.

**Figure 2:**
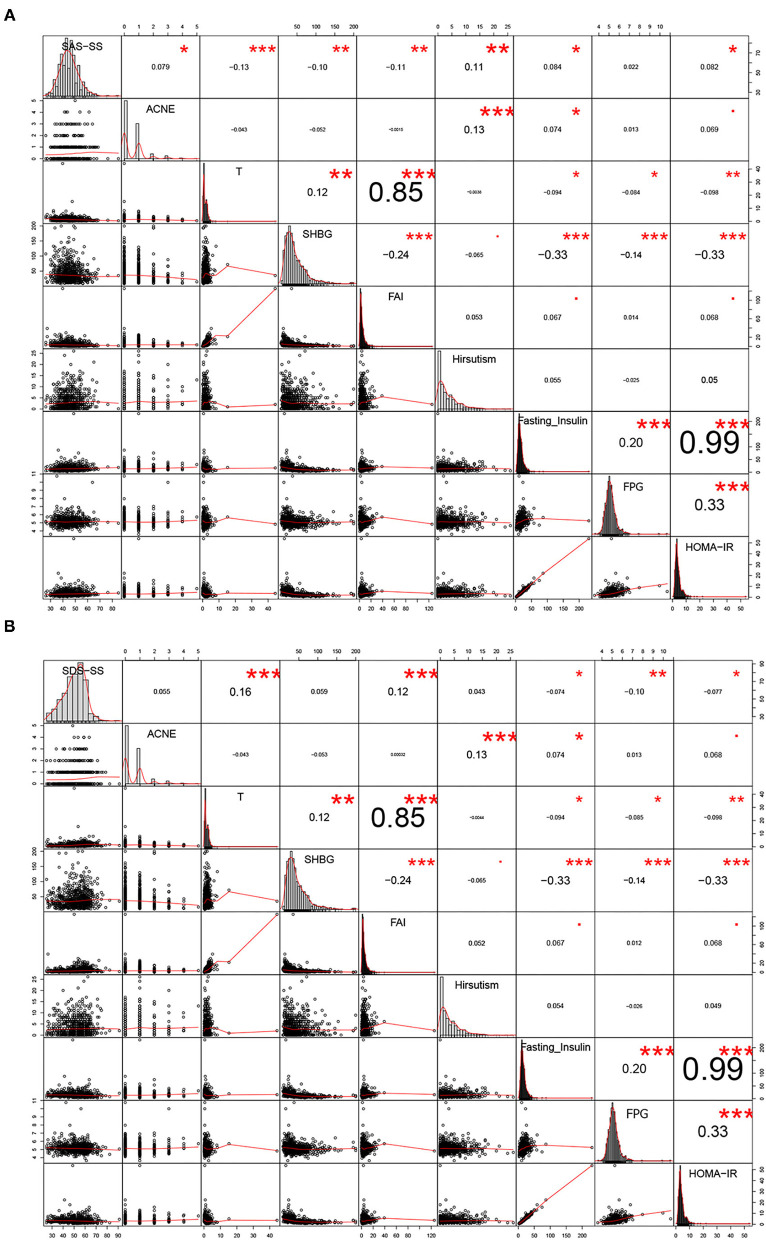
Correlation analyses between anxiety/depression-like behaviors and the 14 clinical parameters. ^*^P < 0.05, ^**^P < 0.01, ^***^P < 0.005.

As shown in Figure 2A, acne, T, SHBG, FAI, hirsutism, fasting insulin, and HOMA-IR were significantly correlated with SAS-SS. T, SHBG, and FAI were negatively correlated (−0.13, −0.10, and −0.11, respectively) (*P* < 0.05), while acne, hirsutism, fasting insulin, and HOMA-IR were positively correlated (0.079, 0.11, 0.084, and 0.082, respectively) (*P* < 0.05). As shown in Figure 2B, both T and FAI were significantly positively correlated with SDS-SS (0.16 and 0.12), while fasting insulin, FBG, and HOMA-IR were significantly negatively correlated with SDS-SS (−0.074, −0.10, and −0.077, respectively). It is worth noting that the factors of age, BMI, W, T, FAI, fasting insulin, and HOMA-IR were significantly correlated with both anxiety-like and depression-like behaviors.

### Linear Regression Analyses Between Anxiety/Depression and Clinical Characteristics

We performed linear regression analysis to evaluate risk factors of anxiety/depression-like behaviors in women with PCOS. As shown in Table 3, FAI, age, BMI, WHR, and hirsutism were significant independent risk factors for anxiety-like behaviors in women with PCOS, with odds ratios of −0.335, −0.258, 0.265, 14.911, and 0.235, respectively (*P* < 0.05). For depression-like behaviors, SHBG, FAI, age, WC, WHR, number of deliveries, fasting insulin, FPG, and HOMA-IR were significant risk factors with odds ratios of 0.203, 1.268, 1.639, −0.403, 46.293, −4.199, −2.805, −11.709, and 10.749, respectively (*P* < 0.05) (Table 4).

**Table 3:** Linear regression analysis between SAS-SS and the clinical parameters of women with PCOS.

**Intercept and variable**	**Prediction model**		
	**β**	**Odd Ratio [95% CI]**	***P*** **-value**
Intercept		33.814 [19.510, 48.117]	0.000
SHBG	−0.045	−0.014 [−0.044, 0.016]	0.364
FAI	−0.248	−0.335 [−0.571, −0.099]	0.006^*^
Age	−0.118	−0.258 [−0.425, −0.091]	0.003^*^
BMI	0.153	0.265 [0.027, 0.503]	0.029^*^
WC	0.019	0.011 [−0.101, 0.124]	0.844
WHR	0.196	14.911 [4.747, 25.074]	0.004^*^
Acne	0.031	0.356 [−0.465, 1.178]	0.395
T	0.149	0.632 [−0.093, 1.357]	0.087
LH/FSH	−0.024	−0.172 [−0.690, 0.346]	0.515
Number of deliveries	0.041	0.699 [−0.584, 1.983]	0.285
Hirsutism	0.102	0.235 [0.070, 0.399]	0.005^*^
Fasting insulin	0.193	0.143 [−0.504, 0.789]	0.665
FPG	−0.020	−0.334 [−2.618, 1.950]	0.774
HOMA-IR	−0.223	−0.673 [−3.398, 2.052]	0.628

*SAS-SS, self-rating anxiety scale standard score; BMI, body mass index; W, waist circumference; WHR, waist-to-hip Ratio; T, testosterone; SHBG, sex hormone-binding globulin; FAI, free androgen index; LH/FSH, the ratio of luteinizing hormone to follicle-stimulating hormone; FPG, fasting plasma glucose; HOMA-IR, homeostatic model assessment of insulin resistance*.

* *P < 0.05*.

**Table 4:** Linear regression analysis between SDS-SS and the clinical parameters of women with PCOS.

**Intercept and variable**	**Prediction model**		
	**β**	**Odds Ratio [95% CI]**	***P*-value**
Intercept		40.430 [−2.151, 83.011]	0.063
SHBG	0.203	0.203 [0.114, 0.292]	<0.001^*^
FAI	0.288	1.268 [0.564, 1.971]	<0.001^*^
Age	0.230	1.639 [1.142, 2.135]	<0.001^*^
BMI	–0.073	−0.412 [−1.120, 0.295]	0.253
WC	–0.210	−0.403 [−0.738, −0.069]	0.018^*^
WHR	0.187	46.293 [16.037, 76.550]	0.003^*^
Acne	0.033	1.244 [−1.201, 3.690]	0.318
T	0.010	0.142 [−2.016, 2.300]	0.897
LH/FSH	–0.023	−0.546 [−2.088, 0.997]	0.488
Number of deliveries	–0.076	−4.199 [−8.021, −0.378]	0.031^*^
Hirsutism	0.030	0.229 [−0.261, 0.719]	0.359
Fasting insulin	−1.168	−2.805 [−4.730, −0.881]	0.004^*^
FPG	–0.217	−11.709 [−18.508, −4.909]	0.001^*^
HOMA-IR	1.094	10.749 [2.637, 18.861]	0.009^*^

*SDS-SS, self-rating depression scale standard score; BMI, body mass index; W, waist circumference; WHR, waist-to-hip Ratio; T, testosterone; SHBG: sex hormone-binding globulin; FAI, free androgen index; LH/FSH: the ratio of luteinizing hormone to follicle-stimulating hormone; FPG, fasting plasma glucose; HOMA-IR, homeostatic model assessment of insulin resistance*.

* *P < 0.05*.

### Subgroup Analysis: Infertile PCOS Patients vs. Fertile PCOS Patients

Of the 369 infertile PCOS patients, 18.42% (68/369) had scores ≥50 indicating anxiety-like behaviors and 70.19% (259/369) had scores ≥53 indicating depression-like behaviors. Of the 433 fertile PCOS patients, 32.56% (141/433) had scores ≥50 indicating anxiety-like behaviors and 36.36% (156/429) had scores ≥53 indicating depression-like behaviors.

**Figure 3:**
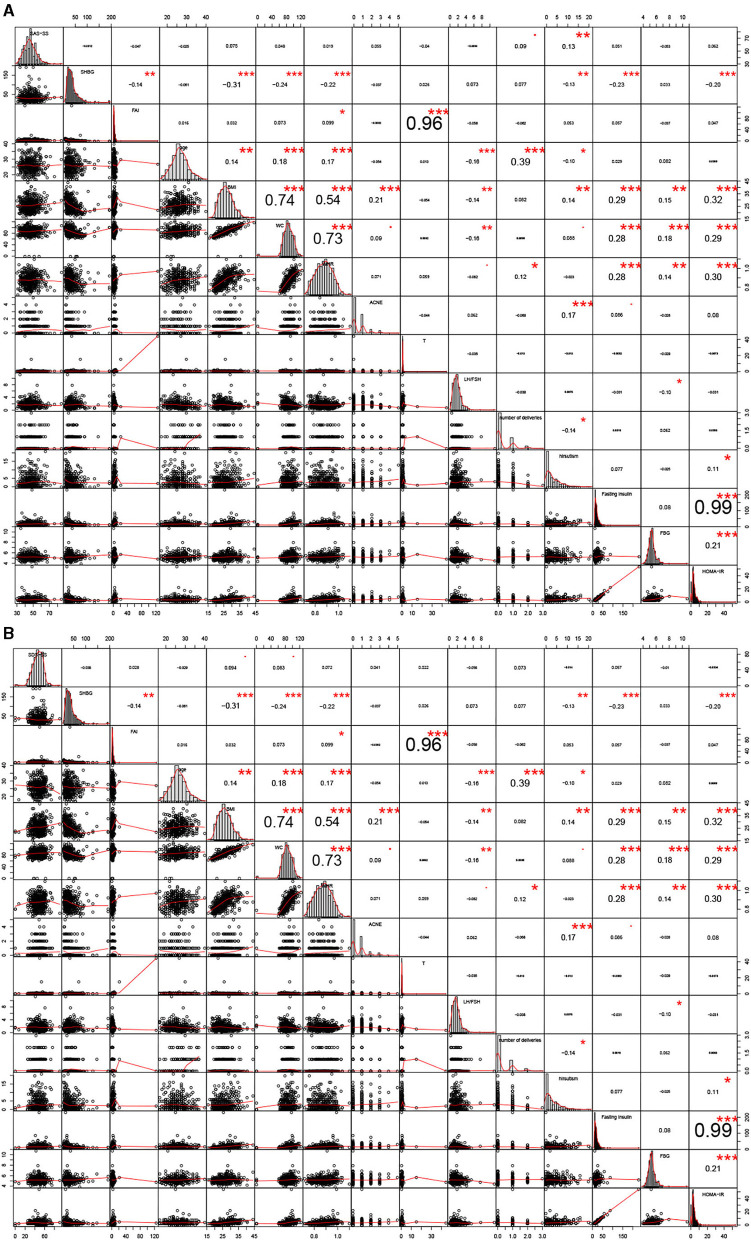
Correlation analyses between anxiety/depression-like behaviors and the 14 clinical parameters in fertile group. ^*^P < 0.05, ^**^P < 0.01, ^***^P < 0.005.

**Figure 4:**
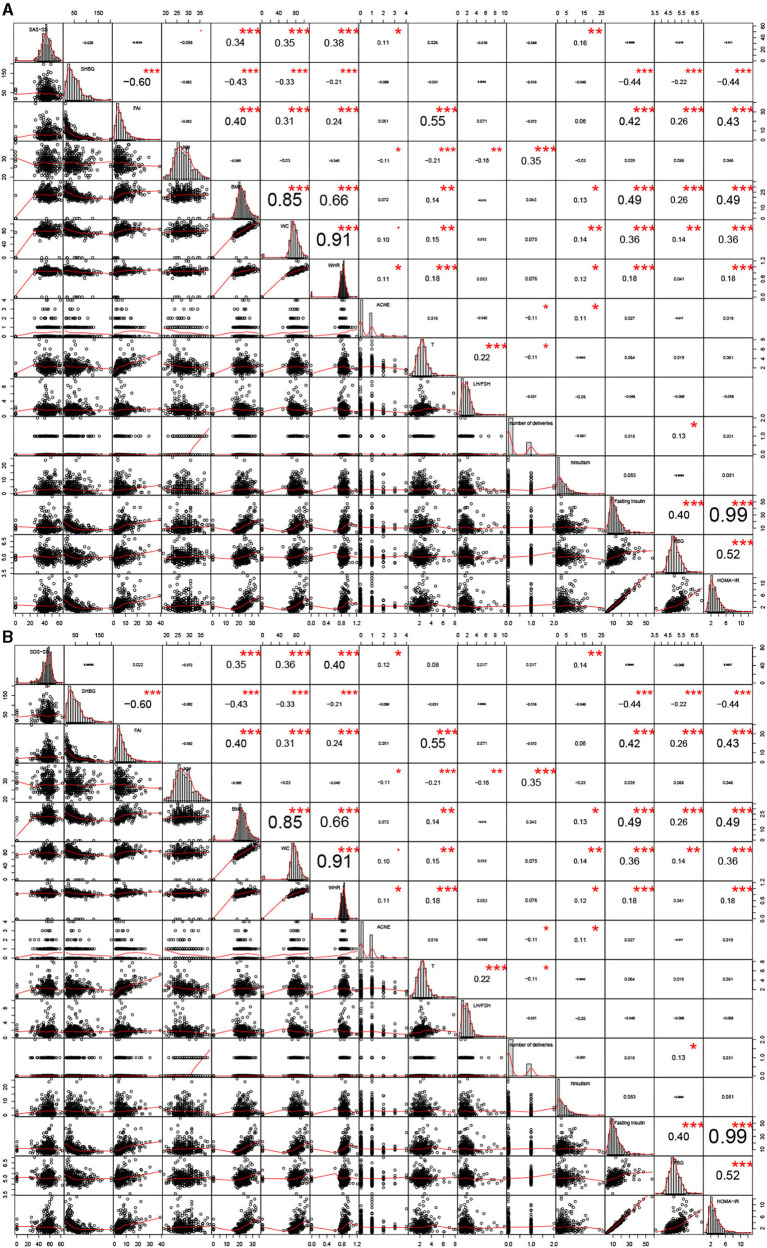
Correlation analyses between anxiety/depression-like behaviors and the 14 clinical parameters in infertile group. ^*^P < 0.05, ^**^P < 0.01, ^***^P < 0.005.

As shown as Supplementary 3, Supplementary Table S1, age, BMI, WC, WHR, acne, T, SHBG, FAI, number of deliveries, fasting insulin, FPG, and HOMA-IR were significantly different (*P* < 0.05) when comparing infertile PCOS patients with fertile PCOS patients. In Figure 3A, number of deliveries and hirsutism were significantly positively correlated with SAS-SS (0.09 and 0.13) in the fertile group. In Figure 3B, BMI and WC were significantly positively correlated with SDS-SS (0.094 and 0.083) in the fertile group. In the infertile PCOS group shown in Figure 4A, age was significantly negatively correlated with SAS-SS (−0.098) while BMI, WC, WHR, acne, and hirsutism were significantly positively correlated with SAS-SS (0.34, 0.35, 0.38, 0.11, and 0.16). In Figure 4B, BMI, WC, WHR, acne, and hirsutism were significantly positively correlated with SDS-SS (0.35, 0.36, 0.40, 0.12, and 0.14).

From the regression analysis results shown in Supplementary 3, Supplementary Tables S2–S4, SHBG was a significant independent risk factor for anxiety-like behaviors in infertile women with PCOS, with an odds ratio of 0.979, and LH/FSH was a significant independent risk factor for depression-like behaviors in fertile women with PCOS with an odds ratio of 0.754.

## Discussion

In this study the prevalence of anxiety-like and depression-like behaviors in women with PCOS was 26.1 and 52.0%, respectively, with the majority of these having a mild degree of anxiety/depression-like behaviors. Among infertile PCOS patients, the prevalence of anxiety-like and depression-like behaviors was 18.42 and 70.19%, compared to 32.56 and 36.36% in fertile PCOS patients, respectively. Anxiety-like behaviors were associated with age, body image-related factors (including BMI and WHR), and hyperandrogenism-related factors (including FAI and hirsutism). Depression-like behaviors were associated with age, body image-related factors (including BMI and WHR), hyperandrogenism-related factors (including FAI and hirsutism), and metabolic factors (including fasting insulin, FBG, and HOMA-IR). Among infertile PCOS patients, anxiety/depression-like behaviors were associated with body image-related factors (including BMI, WC, and WHR) and hyperandrogenism-related factors (including acne and hirsutism). As for fertile PCOS patients, anxiety-like behaviors were associated with number of deliveries and hirsutism while depression-like behaviors were associated with body image-related factors (including BMI and WC).

Different ethnic origin leads to differing manifestations of PCOS. Kakoly et al. reported that Asian, American, and European PCOS patients had 5, 4, and 3-fold increased risks of developing impaired glucose tolerance (3). Uche Ezeh et al. reported that among Americans, African Americans have the greatest BMI and WC, followed by Hispanic White and non-Hispanic White Americans and finally by Asian Americans (26). They reported that African Americans had higher Ferriman–Gallwey scores, which means they had more severe clinical hyperandrogenism but were second in severity in terms of biochemical hyperandrogenism after white Americans (26). In addition, their results showed that African Americans were more likely to have metabolic disorders than white Americans and Asian Americans (26). In all the above aspects, Asian Americans ranked last.

In China, the incidence of PCOS in the Han population is 5.6% (27). Rong Li et al. reported that compared to healthy Chinese women, PCOS patients do not show a higher prevalence of obesity but show a higher prevalence of hyperandrogenism and insulin resistance (IR) (27). Different from American or European women, among Chinese women overweight and obesity are defined as BMI ≥ 24 kg/m^2^ and ≥ 28 kg/m^2^, respectively, and abdominal obesity is defined as WC ≥ 80 cm (28). Under this standard, the prevalence of obesity in PCOS increases with age according to Rong Li (27). In our study, the PCOS patients with IR accounted for 53.62% (444/828), which was similar to the previous report that about 50%−70% of PCOS patients have varying degrees of IR (9). Thus, to a certain degree our results are applicable to the general PCOS population.

Anxiety and depression are the most common mental disorders worldwide. Anxiety disorder is a group of mental disorders characterized by feeling anxious and fearful, while depressive disorders are characterized by grief, loss of interest or happiness, feelings of guilt or low self-worth, disturbed sleep or appetite, fatigue, and poor concentration (29). According to the WHO report about depression and anxiety disorders, the proportions of the global population with depression and anxiety in 2015 were estimated to be 4.4 and 3.6%, respectively (29, 30). For women, the incidences of depression and anxiety globally are 5.1 and 4.6%, respectively. WHO data also showed that for females aged 20–40 years the prevalence of anxiety and depression ranges from 5 to 6% and from 6 to 7%, respectively (29). In our study, the prevalence of anxiety-like and depression-like behavior among all PCOS patients aged 20–40 years rose to 26.1 and 52.0%, respectively, and in the fertile group this rose to 32.56 and 36.36%. Similarly, Cooney et al. reported that the prevalence of anxiety and depression was higher in women with PCOS compared to healthy women (31). For the infertile subgroup, the prevalence of anxiety-like and depression-like behavior was 18.42 and 70.19%. Experiencing infertility brought greater sadness, and this was consistent with previous reports (32).

The pathogenesis of PCOS remains poorly understood (33–36), and PCOS presents as a diverse and complicated syndrome involving reproductive dysfunction (infertility and menstrual irregularity), metabolic problems (dyslipidemia, obesity, increased insulin levels, and glucose intolerance), cardiovascular problems (coronary heart disease), psychological disorders (anxiety and depression), and sexual dysfunction (37–41). In our study, we mainly focused on the psychological impact of PCOS. Previous studies have shown that PCOS patients tend to be under greater psychological pressure because they often experience more concern about body image-related factors as well as more worries about infertility (13, 42). Such negative thoughts can bring about negative emotions such as anxiety, worry, fear, depression, and low self-esteem (42). In this study, we similarly found that body image-related factors, including BMI, WHR, and hirsutism, were related to the occurrence of anxiety-like behaviors and that BMI, WC, and WHR were related to the occurrence of depression-like behaviors. Also, regardless of fertility status BMI and WC were related to the occurrence of depression-like behaviors. As for the occurrence of anxiety-like behaviors, acne and hirsutism were related to the infertile group while only hirsutism was related to the fertile group. Others have demonstrated that a higher BMI, which is known to be independently associated with PCOS, is linked to greater dissatisfaction with one's appearance (43, 44). Some researchers have also suggested recently that PCOS symptoms such as changes in body size and appearance might result in reduced quality of life and reduced psychological, social, and sexual well-being (45). Acne and hirsutism are prevalent in women with PCOS (46), and some studies suggest that these features are associated with more severe anxiety and depression symptoms in women with PCOS (47). Taken together, long-term negative feelings about one's appearance likely lead to a high prevalence of poor mental health such as anxiety or depression in women with PCOS.

One point worth noting was that age, body image related factors and hyperandrogenism related factors are related to the occurrence of anxiety/depression-like behaviors, the correlation coefficients of two groups were completely opposite. The PCOS patients with older age, lower BMI, lighter acne and IR but higher T tended to correlated with depression-like behaviors, while younger PCOS patients were opposite, as shown in Supplementary 3, Supplementary Table S6. We have not found relevant literature so far. Future studies needed to be undertaken to explain it.

Hyperandrogenism is another important aspect of PCOS that should be taken into consideration when exploring psychological outcomes, and we found that FAI was a significant independent factor for these two behaviors and was positively correlated with depression-like behaviors and negatively correlated with anxiety-like behaviors. Clinical hyperandrogenism, i.e., hirsutism, is present in 6.1–10.0% of women with PCOS in China, but the prevalence of biochemical hyperandrogenism could be as high as 21.1% (48, 49). A large meta-analysis reported that both clinical and biochemical hyperandrogenism are related to the prevalence of anxiety/depression-like behaviors in women with PCOS (31) and the specific manifestations of clinical hyperandrogenism—hirsutism and acne—likely lead to an increased prevalence of depression and anxiety in this population (50). Ferriman–Gallwey scores, which are used to evaluate hirsutism, were reported to be increased in women with PCOS with anxiety and depression symptoms, and free T levels were higher in women with PCOS and anxiety compared to those without anxiety (51). In our study, FAI was significantly lower in the anxiety-like group and significantly higher in the depression-like group when compared to the normal-scoring groups.

In our study, fasting insulin, FBG, and HOMA-IR were significantly negatively correlated with SDS-SS in women with PCOS, and metabolic disorder was an important factor affecting depression-like behaviors. It was reported previously that depression is linked to blood insulin tested at 2 h in a glucose tolerance test (52). However, more high-quality research should be carried out in the future.

The strength of our study is that our data on the prevalence of anxiety-like and depression-like behaviors was in a relatively large sample of women with PCOS from an ethnic background for which there are limited relevant data. A limitation of our study lies in potential selection/recruitment bias of the participants because this was a secondary analysis of three previous studies performed to study various effects of acupuncture in women with PCOS. Based on the inclusion criteria of the initial studies, some of the recruited women with PCOS had IR and some of them had infertility. Also, only Chinese women were involved in this study. Another limitation is that both anxiety-like and depression-like behaviors were self-reported. Also, some factors such as smoking and quality of sleep were not considered, and this might make our results less reliable.

In conclusion, the prevalence of anxiety-like and depression-like behaviors in women with PCOS was 26.1 and 52.0%, respectively. In fertile PCOS patients, the prevalence was 32.56 and 36.36%, respectively, and in infertile PCOS patients the prevalence was 18.42 and 70.19%. Age, BMI, WHR, and FAI were associated with both anxiety and depression-like symptoms, while hirsutism was an important factor for anxiety-like behaviors and fasting insulin, FBG, and HOMA-IR were important factors for depression-like behaviors. Body image-related factors and hyperandrogenism-related factors were related to both anxiety-like behaviors and depression-like behaviors in both infertile and fertile PCOS patients.

## Data Availability Statement

The data analyzed in this study is subject to the following licenses/restrictions: The datasets generated and/or analyzed during the current study are not publicly available but are available from the corresponding author on reasonable request. Requests to access these datasets should be directed to doctorhongxia@126.com.

## Ethics Statement

The studies involving human participants were reviewed and approved by the Ethics Committee of the First Affiliated Hospital of Guangzhou Medical University (No. 2013039, No. 2015010, No. 2014018). The patients/participants provided their written informed consent to participate in this study. Written informed consent was obtained from the individual(s) for the publication of any potentially identifiable images or data included in this article.

## Author Contributions

HM had full access to all of the data in the study and takes responsibility for the integrity of the data and the accuracy of the data analysis. HL: project development, data collection, data analysis, and manuscript writing. ML, DZ, and HM: project development, data collection, manuscript writing, and revision. EN, JIL, and JUL: data collection and manuscript revision. YS, CZ, XW, ZM, and MO: data collection. All authors contributed to the article and approved the submitted version.

## Conflict of Interest

The authors declare that the research was conducted in the absence of any commercial or financial relationships that could be construed as a potential conflict of interest.

## Publisher's Note

All claims expressed in this article are solely those of the authors and do not necessarily represent those of their affiliated organizations, or those of the publisher, the editors and the reviewers. Any product that may be evaluated in this article, or claim that may be made by its manufacturer, is not guaranteed or endorsed by the publisher.
