# Supplementary material for: The Prevalence and Factors Associated With Anxiety-Like and Depression-Like Behaviors in Women With Polycystic Ovary Syndrome
Source: Front Psychiatry. 2021 Oct 20;12:709674. doi: 10.3389/fpsyt.2021.709674 (PMC8563587; doi:10.3389/fpsyt.2021.709674)
Supplement: Supplementary file 1 [file Data_Sheet_1.docx]

Supplement 1.Specific Message of Ethics approval

1.Ethics approval of *The Effect of Acupuncture on Insulin Sensitivity Polycystic Ovary Syndrome* (2015)

This study was approved by the ethics committee of the First Affiliated Hospital of Guangzhou Medical University (No. 2013039).

2.Ethics approval of *The Effect of Acupuncture on Insulin Sensitivity of Women with Polycystic Ovary Syndrome and Insulin Resistance: A Randomized Controlled Trial* (2016)

This study has been approved by the Ethics Committee of the First Affiliated Hospital of Guangzhou Medical University, Xuzhou Maternity and Child Health Hospital, and Hexian Memorial Affiliated Hospital of Southern Medical University. (Reference: 2015010)

3.Ethics approval and consent to participate of *The Effect of Acupuncture Pre-treatment Combined with Letrozole on Live Birth in Infertile Women with Polycystic Ovary Syndrome: a Randomized Controlled Trial* (2017)

This study had been approved by the ethics committee of the First Affiliated Hospital of Guangzhou Medical University, Xuzhou Maternity & Child Health Hospital, Dalian Municipal Women and Children’s Medical Center, Guangdong Women and Children’s Hospital, and Hexian Memorial Affiliated Hospital of Southern Medical University (Reference: 2014018). All participants signed the informed consent form prior to participation.
