# Supplementary material for: The Prevalence and Factors Associated With Anxiety-Like and Depression-Like Behaviors in Women With Polycystic Ovary Syndrome
Source: Front Psychiatry. 2021 Oct 20;12:709674. doi: 10.3389/fpsyt.2021.709674 (PMC8563587; doi:10.3389/fpsyt.2021.709674)
Supplement: Supplementary file 2 [file Data_Sheet_2.docx]

**Supplement.2 Inclusion criteria and Exclusion Criteria of three RCT studies**

***1.The Effect of Acupuncture on Insulin Sensitivity Polycystic Ovary Syndrome* (ClinicalTrials.gov NCT 02026323)**

Inclusion criteria

1. Age between 18 and 40 years.

2. BMI≥18.5 kg/m2.

3. Presence of PCOS as defined by the Rotterdam criteria and including at least two of the following three features:

▸Oligomenorrhoea or amenorrhoea: Oligomenorrhoea is defined as an intermenstrual interval >35 days or <8 menstrual bleedings in the past year. Amenorrhoea is defined as complete cessation of menstrual cycles for 6 months or more when a patient has previously had regular cycles and for 12 months or more when the patient has had irregular cycles.

▸ Clinical or biochemical hyperandrogenism: Biochemical hyperandrogenaemia is defined as a

total serum testosterone concentration above 60 ng/dL,26 and clinical hyperandrogenism is defined as a Ferriman-Gallwey (FG) score ≥5, in mainland China.

▸ Polycystic ovary morphology: This is defined as ≥12 antral follicles (2–9 mm in diameter) or an ovarian volume >10 mL on transvaginal scanning.

4. Presence of IR as defined by the homeostatic model assessment (HOMA-IR: (fasting insulin (μU/mL)×fasting glucose (mmol/L))/22.5). A value ≥2.14 will be considered to be indicative of IR.

5. No desire to bear children and having used barrier methods of contraception for 1 year.

6. Willingness to sign the consent form.

Exclusion criteria

1. Having other endocrine disorders such as hyperprolactinaemia (defined as two prolactin levels measured at least 1 week apart of 25 ng/mL or greater or as determined by local normative values), non-classic congenital adrenal hyperplasia (17-hydroxyprogesterone <3 nmol/L), or androgen-secreting tumours.

2. Follicle-stimulating hormone (FSH) levels >15 mIU/mL. A normal level within the last year is adequate for entry.

3. Uncorrected thyroid disease defined as thyroidstimulating hormone (TSH) <0.2 mIU/mL or TSH>5.5 mIU/mL. A normal level within the last year is adequate for entry.

4. Patients with type I diabetes mellitus or type I and type II who are receiving antidiabetic medications such as insulin, thiazolidinediones, acarbose, sulfonylureas, or other medications that are likely to confound the effects of the study. Patients currently receiving metformin for a diagnosis of type I or type II diabetes or for PCOS are also specifically excluded.

5. Suspected Cushing’s syndrome.

6. Use of hormones or other medications in the past 3 months, including Chinese herbal prescriptions, which might affect the outcome.

7. Pregnancy within the past 6 weeks.

8. Postabortion or post partum within the past 6 weeks.

9. Breast feeding within the past 6 months.

10. Receiving acupuncture treatment related to PCOS within the past 2 months.

11. Having undergone a bariatric surgery procedure within the past 12 months or being in a period of acute weight loss.

12. Having known congenital adrenal hyperplasia.

13. Lack of written consent to participate in the study. Eligible participants will be recruited and categorized according to their BMI as normal weight (BMI=18.5–23 kg/m2) or overweight/obese (BMI≥23 kg/m2).

***2.The Effect of Acupuncture on Insulin Sensitivity of Women with Polycystic Ovary Syndrome and Insulin Resistance: a Randomized Controlled Trial*(ClinicalTrials.gov, NCT 02491333)**

Inclusion criteria

1. Women aged between 20 and 40 years.
 2. Confirmed diagnosis of PCOS according to the Rotterdam criteria: oligomenorrhoea (an intermenstrual interval>35 days or <8 cycles in the past year) or amenorrhoea (an intermenstrual interval>90 days) together with polycystic ovarian morphology, that is, the presence of ≥12 antral follicles (≤9 mm) and/or ovarian volume >10 mL on transvaginal scanning, and/or clinical or biochemical hyperandrogenism. Clinical hyperandrogenism in China is defined as a Ferriman-Gallwey (FG) score ≥5, and biochemical hyperandrogenism is defined as total testosterone (T)>2.6 nmol/L and free testosterone ≥6.0 pg/mL.

3. A husband whose sperm concentration meets the WHO standards (2010) of ≥15×106/mL and a total motility of ≥40% or a total motile sperm count of ≥9 million.

4. At least one patent tube shown by hysterosalpingogram or diagnostic laparoscopy within 3 years if the patient does not have a history of abortion or pelvic operation. If the patient has a history of pregnancy and no history of pelvic operation within the past 5 years, she is not required to undergo a tubal patency test.

Exclusion criteria

1. Exclusion of other endocrine disorders:

▸ Patients with hyperprolactinaemia (defined as 2 prolactin (PRL) levels of ≥25 ng/mL at least 1 week apart or as determined by local normative values). The goal of eliminating patients with documented hyperprolactinaemia is to decrease the heterogeneity of the PCOS population.

These patients might be candidates for ovulation induction with alternate regimens (dopamine

agonists). A normal level within the past year or being on treatment is adequate for entry.

▸ Patients with follicle-stimulating hormone (FSH) levels >15 mIU/mL. A normal level within the past year is adequate for entry.

▸ –Patients with uncorrected thyroid disease (defined as thyroid-stimulating hormone (TSH) <0.2 or >5.5 mIU/mL). A normal level within the past year is adequate for entry.

▸ Patients diagnosed with type I or type II diabetes who are poorly controlled (defined as a glycated haemoglobin (HbA1c) level >7.0%) or patients receiving antidiabetic medications such as insulin, thiazolidinediones, acarbose or sulfonylureas that are likely to confound the effects of electroacupuncture. Patients currently receiving metformin XR (extended release) for a diagnosis of type I or type II diabetes or for PCOS are also specifically excluded.

▸ Patients with suspected Cushing’s syndrome.

2. Use of hormonal or other medication, including Chinese herbal prescriptions, in the past 2 months that might affect the outcome of the study treatment.

3. Acupuncture in the past 2 months.

4. Pregnancy within the past 6 weeks.

5. Abortion or having given birth in the past 6 weeks.

6. Breast feeding within the past 6 months.

7. Not willing to give written consent to the study.

8. Patients enrolled simultaneously in other investigative studies that require medications, prohibit the use of the study medications, limit intercourse or otherwise prevent compliance with the study protocol.

9. Patients who anticipate taking longer than a 1 month break from treatments during the study protocol.

10. Additional exclusion criteria:

A. Patients with a suspected adrenal or ovarian tumour that is secreting androgens.

B. Couples with previous sterilisation procedures (vasectomy, tubal ligation) that have been reversed. The prior procedure might affect the study outcomes, and patients with both a reversed sterilisation procedure and PCOS are rare enough that exclusion should not adversely

affect recruitment.

C. Participants who have undergone a bariatric surgery procedure in the recent past (<12 months) and are in a period of acute weight loss or who have been advised against pregnancy by their bariatric surgeon.

D. Patients with untreated or poorly controlled hypertension defined as a systolic blood pressure of 160 mm Hg or a diastolic blood pressure of 100 mm Hg obtained on two occasions at least 60 min apart.

E. Patients with known congenital adrenal hyperplasia.

F. Patients on oral contraceptives, depot progestins or hormonal implants (including Implanon). A 2-month washout period will be required prior to screening for patients on these agents. Longer washouts might be necessary for certain depot contraceptive forms or implants, especially when the implants are still in place. A 2-month washout will be required for patients on oral cyclic progestins.

G. Patients with liver (LR) disease defined as aspartate aminotransferase (AST) or alanine aminotransferase (ALT) >2 times normal or total bilirubin >2.5 mg/dL or patients with renal

disease defined as blood urea nitrogen (BUN) >30 mg/dL or serum creatinine >1.4 mg/dL.

H. Patients with significant anaemia (haemoglobin <10 g/dL).

I. Patients with a history of deep venous thrombosis, pulmonary embolus or cerebrovascular accident.

J. Patients with known heart disease that is likely to be exacerbated by pregnancy.

K. Patients with a history of, or suspected, cervical carcinoma, endometrial carcinoma or breast carcinoma. A normal Pap smear or thinprep cytologic test (TCT) result will be required for women 21 years and older.

***3.The Effect of Acupuncture Pre-treatment Combined with Letrozole on Live Birth in Infertile Women with Polycystic Ovary Syndrome: a Randomized Controlled Trial* (ClinicalTrials.gov NCT 02491320)**

(1)Chinese women aged from 18 to 40 years.

(2)Body mass index (BMI) ≥18.5 kg/m2.

(3)Confirmed diagnosis of PCOS according to the Rotterdam criteria in 2003 including at least two of the following three features: (1) oligo- (an intermenstrual interval >35 days or <8 cycles in the past year) or amenorrhea (an intermenstrual interval >90 days), and/or (2) polycystic ovarian morphology, i.e., presence of >12 antral follicles (≤9 mm) and/or ovarian volume >10 ml on ultrasonography scanning, and/or (3) clinical and/or biochemical hyperandrogenism. Clinical hyperandrogenism on the Chinese Mainland is defined as a Ferriman-Gallwey (FG) score ≥5 ; biochemical hyperandrogenism is total testosterone (T) >2.6 nmol/l and free testosterone ≥6.0 pg/ml .

(4)Presence of IR as defined by the homeostatic model assessment—HOMA-IR: [fasting insulin (μU/ml) × fasting glucose (mmol/l)] / 22.5). A value ≥2.14 is considered to be indicative of IR .

(5)No immediate fertility wish and willingness to use barrier contraceptive methods for 7 months.

(6)Willingness to sign the consent form.

Exclusion Criteria

(1)Exclusion of other endocrine disorders:

(1)Uncorrected thyroid disease (defined as thyroid stimulating hormone (TSH) < 0.2 mIU/ml or >5.5 mIU/ml, triiodothyronine (T3) < 1.4 nmol/l or >2.2 nmol/l, and free thyroxine (T4) < 10 pmol/l or >23 pmol/l). A normal level within the last year is adequate for entry.

(2)Poorly controlled type I or type II diabetes (defined as a glycosylated hemoglobin (HbA1c) level >7.0%) or patients receiving antidiabetic medications such as insulin, thiazolidinediones, acarbose, or sulfonylureas likely to confound the effects of the study medication; patients currently receiving metformin XR (extended release) for a diagnosis of type I or type II diabetes or for PCOS are also excluded.

(3)Cushing’s syndrome (defined as an archetype of metabolism syndrome. High glucocorticoid levels lead to muscle, liver, and adipocyte insulin resistance; 17-hydorxycorticosteroids >55 umol/ 24 h or urinary-free cortisol >304 nmol/24 h).

(4)Congenital adrenal hyperplasia (defined as patients with known 21-hydroxylasedeficiency or other enzyme deficiency leading to the phenotype of congenital adrenal hyperplasia; 17-oh progesterone >10 ng/ml in adrenocorticotropic hormone 1-24 h excited test (after 60 min).

(5)Suspected androgen-secreting adrenal or ovarian tumor.

(2)Use of hormonal or other medication including Chinese herbal prescriptions, which may affect the outcome of the last 2 months.

(3)Receiving acupuncture in the past 2 months.

(4)Within 6 weeks of pregnancy.

(5)Post-abortion or postpartum within the past 6 weeks.

(6)Breastfeeding within the last 4 months.

(7)Not willing to give written consent to the study.

(8)Having a bariatric surgery procedure within the past 12 months or being in a period of acute weight loss.

(9)Additional exclusion criteria include:

a. Patients on oral contraceptives, depot progestin, or hormonal implants (including Implanon). A 2-month washout period will be required prior to screening for patients on these agents. Longer washouts may be necessary for certain depot contraceptive forms or implants, especially where the implants are still in place. A 1-month washout will be required for patients on oral cyclic progestin.

b. Heart disease.

c. Patients with a history of or suspected cervical carcinoma, endometrial carcinoma, or breast carcinoma.

d. Patients enrolled in other studies that require medications.

e. Patients taking longer than a 1-month break during the protocol should not be enrolled.
